# Alleviation of depression-like behavior in a cystic fibrosis mouse model by Hdac6 depletion

**DOI:** 10.1038/s41598-020-73298-4

**Published:** 2020-10-01

**Authors:** Deborah A. Corey, Sharon M. Rymut, Thomas J. Kelley

**Affiliations:** Department of Genetics and Genome Sciences, Case Western Reserve University, 833 BRB, 10900 Euclid Avenue, Cleveland, OH 44106-4948 USA

**Keywords:** Depression, Preclinical research

## Abstract

Cystic fibrosis (CF) patients experience heightened levels of anxiety and depression. Stress from dealing with chronic disease and rigorous treatment regimens certainly are primary contributors to these outcomes. We previously have demonstrated that microtubule alterations in CF are linked to a number of CF phenotypes including growth regulation and inflammatory responses to airway bacterial challenge. Deletion of histone deactelyase 6 (HDAC6), a cytosolic deacetylase that regulates tubulin acetylation, in CF mice restores growth and inflammatory phenotypes to wild type (WT) profiles. In this study, the hypothesis that Hdac6 depletion in CF mice would impact behaviors since Hda6 inhibition has been previously reported to have anti-depressive properties. Data demonstrate that CF mice exhibit reduced activity and reduced open arm time in an elevated plus maze test which can be consistent with anxiety-like behavior. CF mice also exhibit depression-like behaviors compared to WT mice in an age dependent manner. By eight weeks of age, CF mice exhibit significantly more immobile time in the tail-suspension test, however, Hdac6 depletion reverses the depressive phenotype. These data demonstrate that loss of CFTR function may predispose patients to experience depression and that this behavior is Hdac6 dependent.

## Introduction

Loss of function of the cystic fibrosis transmembrane conductance regulator (CFTR) chloride channel causes varied physiological malfunctions including chronic lung inflammation^1^, CF-related diabetes^2^, impaired growth and weight^3,4^ and obstructive bowel disease^5^ all contributing to a disease that is difficult to manage. While advances in health care have prolonged lifespan, stresses related to early mortality, repeated hospitalization and management of a chronic disease contributes to poor mental health in CF patients including depression and anxiety^6–10^.

Research has shown that depression and anxiety are more prevalent in cystic fibrosis patients when compared to the general population. The International Depression Epidemiological Study (TIDES) conducted across nine countries showed that depression and anxiety were elevated in 10% adolescent and 19% adult CF patients, while anxiety was also found to be increased in 22% and 32% of adolescents and adults, respectively^11^. Depression and anxiety can have adverse effects on CF patients in that they are less likely to be compliant with treatment, have worse lung function, and have increased number of hospital visits^12^. Therefore, a corresponding link between loss of CFTR function and depression and anxiety needs to be examined in order to uncover potential therapies thereby improving the quality of life for CF patients. Earlier mouse studies by Bregman and Fride demonstrate that CF mice exhibit anxiety-like behavior that is sensitive to Δ9-tetrahydrocannabinol treatment^13^. These findings suggest an innate biological mechanism for heightened anxiety in CF.

Normal neuronal functioning depends on microtubule-related dynamics and disruption of microtubule structure and function has been implicated in a number of diseases including Alzheimer’s, Amyotrophic Lateral Sclerosis and Parkinson’s Disease^14^. Alterations in microtubule binding proteins such as Tau, which is involved in stabilizing microtubules, can also lead to impaired neurological functioning. Further, Tau interacts with microtubule motor proteins and regulates cargo transport along the axon^15–17^. We have demonstrated specific microtubule disruption in CF epithelial cells leading to the hypothesis that manipulation of microtubule regulation in CF mice may impact behaviors.

Mechanistically, we hypothesize that histone deacetylase 6 (HDAC6) inhibition would benefit behavioral changes associated with CF. HDAC6 is a member of a ubiquitous, large family of enzymes that remove acetyl groups from lysine residues in proteins and acts on non-histone proteins including alpha tubulin^18^. We have shown slower microtubule reformation rates, as well as decreased acetylated microtubule levels in CF cells and tissues indicating inherent changes to microtubule regulation in the absence of CFTR^19–21^. Inhibition of HDAC6 in CF cells restores intracellular transport and inflammatory signaling regulation^19^. Examining the importance of HDAC6 in vivo in a CF context, we demonstrated that depletion of Hdac6 expression from a CF mouse model increased CF mouse linear growth, increased weight gain, and restored the ability to store fat^22^. We also demonstrated the depletion of Hdac6 in CF mice normalizes the inflammatory response and the ability to clear bacteria in a murine airway infection model^23^. The restoration of linear growth and the increase in insulin-like growth factor-1 (IGF-1) in CF mice lacking Hdac6 expression suggests that the hypothalamus-pituitary axis is being affected by Hdac6 depletion indicating a direct neurological impact^24,25^. Therefore, depletion of Hdac6 may also improve behavior phenotypes in CF mice. Evidence for HDAC6 control of behavior has been shown. Fukada et al. showed that in behavioral studies, Hdac6 deficient mice were less anxious, more hyperactive and showed less depressive traits then WT mice^26^.

The behavior traits associated with HDAC6 expression strongly suggest that HDAC6 is expressed in the brain. The study described above by Fukada et al. demonstrates that HDAC6 is strongly expressed in serotonergic neurons in the Raphe nuclei^26^. There is also expression shown in the hippocampus and cerebral cortex, with weaker staining in other regions^26^. Strebl et al. have mapped HDAC6 expression in rodent on non-human primate brains using fluorescently labeled bavarostat, an HDAC6 selective inhibitor^27^. These data demonstrate that HDAC6 is in the brain and in regions associated with behavior regulation.

In this manuscript, we demonstrate a higher incidence of depression-like behavior in an F508del/F508del (CF) mouse model and that knocking out Hdac6 in this CF mouse (DKO) corrects the depression-like phenotype in an age dependent manner.

## Results

### Anxiety-related behavior analysis

#### Elevated plus maze

The elevated plus maze is a standard test for measuring anxiety in rodents^28^. Wild-type, CF, Hdac6 knockout (HDA) and CF/Hdac6 double knockout mice (DKO) were allowed to move freely through out an elevated plus maze for five minutes. The movements and behavior of the mice were video recorded and videos were analyzed. Movement throughout the maze was analyzed by two different means: total distance traveled and location preference.

#### Distance traveled

**Figure 1:**
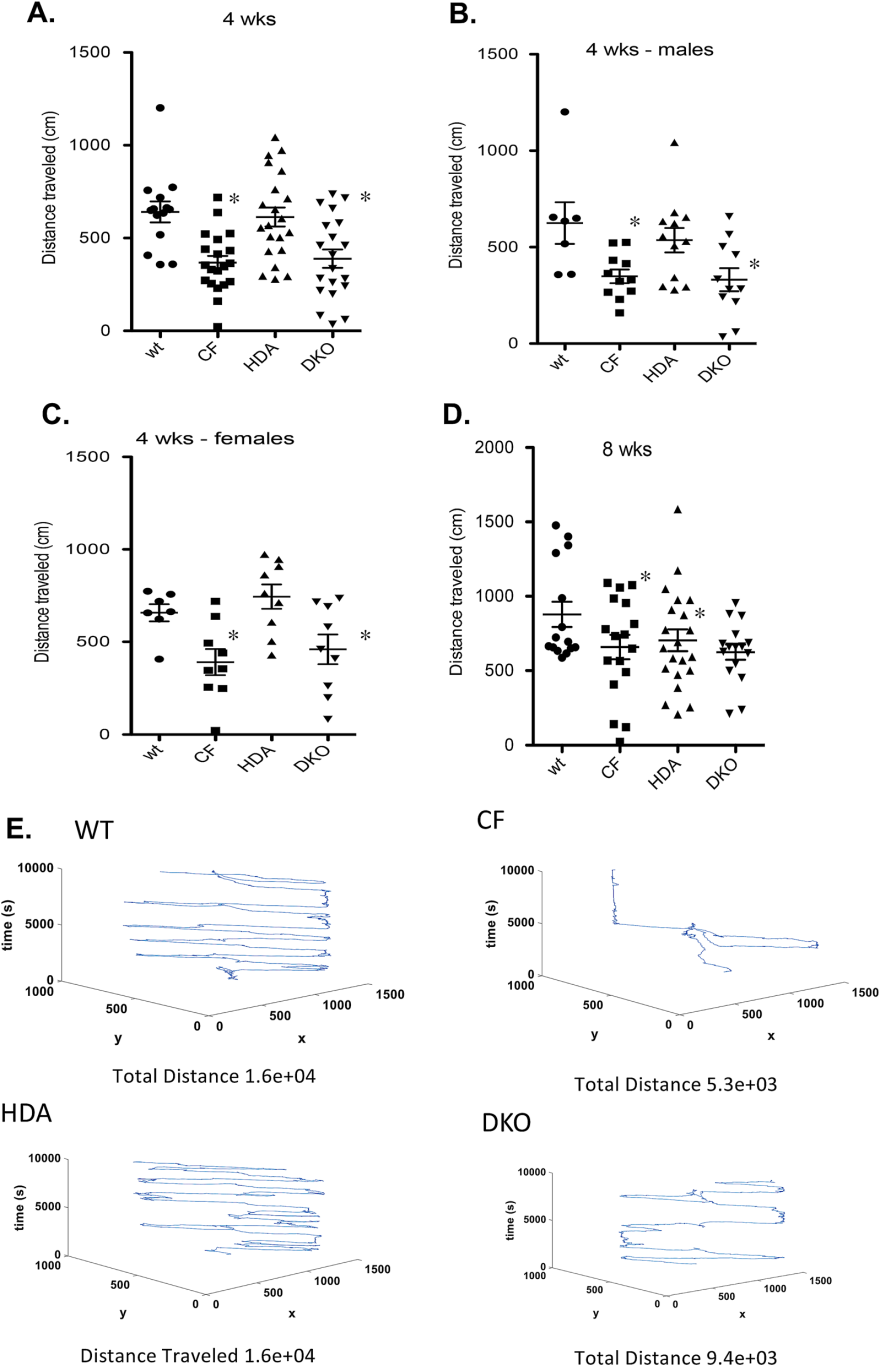
Total distance traveled in elevated-plus maze. Total distance traveled (cm) in 4-week old (**A**) and 8-week old (**D**) grouped male and female wild type (WT), F508del/F508del (CF), Hdac6-/-(HDA), and CF/HDA (DKO) mice. Responses are separated to male (**B**) and female (**C**) 4-week old mice. (**D**) Movement traces generated by IdTracker are shown for WT, CF, HDA, and DKO mice. Significance determine by ANOVA with Newman–Kuels post-hoc test to compare groups; *p < 0.05 compared to WT control group. Replicates for each study are: (**A**) WT = 10, CF = 20, HDA = 20, DKO = 20; (**B**) WT = 7, CF = 11, HDA = 12, DKO = 11; (**C**) WT = 7, CF = 9, HDA = 9, DKO = 9; (**D**) WT = 15, CF = 17, HDA = 21, DKO = 15.

Total distance moved was assessed in CF and WT mice at both 4- and 8-weeks of age to assess activity level. These time points are based on our previous results examining growth regulation in CF mice and the effect of Hdac6 depletion on that growth. In DKO mice, CF-like growth was seen at 4-weeks of age and a rapid recovery to WT size was observed by 8-weeks of age^22^. These results indicate that a significant change in regulation likely occurs post-puberty in DKO mice in a CF model. Therefore, behavior at these time points was examined. Hdac6 depletion had no effect on growth until after 4 weeks of age. Our date with depression reversal are consistent with those findings suggesting that neurological effects of Hdac6 are age dependent. CF mice traveled significantly less throughout the elevated plus maze when compared to WT controls at 4 weeks of age (WT: 641.2 ± 56.6 cm; CF: 367.7 ± 36.4 cm). Depletion of Hdac6 expression did not normalize total distance traveled in 4-week old DKO mice (DKO: 389.1 ± 49.7 cm) (Fig. 1A). To determine if gender had a bearing on the results, we stratified the age groups into males and females. As seen in the combined gender analysis, 4-week old CF males exhibited significantly reduced total distance traveled when compared to wild-type (WT: 625.1 ± 107.8 cm; CF 348.9 ± 35.2 cm), which again was not normalized by the removal of Hdac6 (DKO: 331.3 ± 60.0 cm) (Fig. 1B). The total distance traveled for the female mice show similar distributions, also with no significant effect of Hdac6 depletion (Fig. 1C). By 8-weeks of age, similar trends in total distance traveled are seen but no significant differences are observed between groups (Fig. 1D). Total distance movement as represented is seen in Fig. 1E. These results demonstrate reduced activity in CF mice is more pronounced at 4-weeks of age compared to 8-weeks. The depletion of Hdac6 expression in CF mice does not improve activity level at either age.

#### Location preference

**Figure 2:**
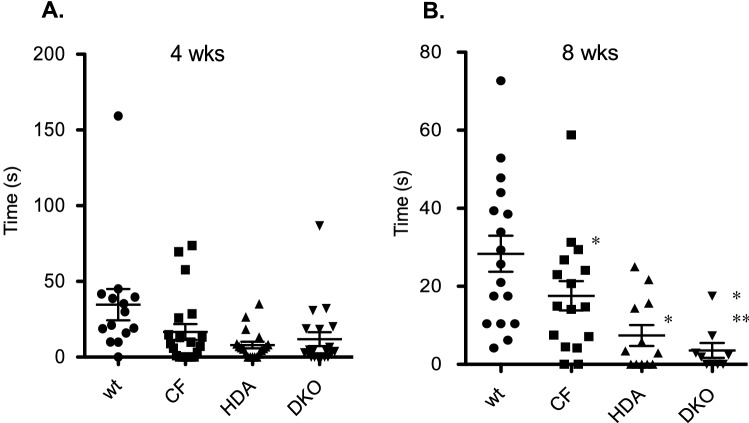
Open arm time in elevated-plus maze. Time spent in open-arm in 4-week old (**A**) and 8-week old (**B**) grouped male and female wild type (WT), F508del/F508del (CF), Hdac6-/- (HDA), and CF/HDA (DKO) mice. Significance determine by ANOVA with Newman–Kuels post-hoc test to compare groups; *p < 0.05 compared to WT control group, **p < 0.05 compared to CF test group. Replicates are 13 mice for each group at 4-weeks of age and n = 16 for each group at 16 weeks of age.

To further examine anxiety in CF mice compared to WT we also analyzed their location preference in the maze. Mice that are demonstrating anxiety-like behaviors will be less likely to enter into an open, unprotected arm of the EPM. The location preference of the mice was recorded using EPMscore. Mice were determined to be in an arm when > 50% of the length of their body entered that field. CF mice at 4-weeks of age display significantly reduced open arm time (OAT) suggesting the presence of more anxiety compared to the WT counterparts (WT: 34.6 ± 10.3 s; CF: 16.6 ± 5.2 s), however, loss of Hdac6 expression did not significantly change OAT in DKO mice compared to CF mice at 4-weeks of age (DKO: 11.8 ± 4.6 s) (Fig. 2A). CF mice show similar reduced OAT compared to WT mice at 8-weeks. Interestingly, depletion of Hdac6 expression further reduces this time in both WT and CF backgrounds (WT: 28.3 ± 4.6 s; CF: 17.6 ± 3.8 s; DKO: 3.6 ± 1.9 s) (Fig. 2B). These data suggest that anxiety-like behavior may actually increase in the absence of Hdac6. These findings are in contradiction to Fukada et al., where that study demonstrates decreased anxiety levels in Hdac6 KO mice^26^. The Fukada study used mice 3–12 months of age, significantly older than the 4–8 week-old mice we study to correlate with our previous growth studies. Age can have a significant influence on anxiety and stress responses and we do not have corresponding data to compare. There are no significant gender differences in responses (data not shown).

Rearing behavior, a variant of the search phase of exploratory behavior, was also evaluated between groups. At 4 weeks of age, no difference in rearing was observed. By 8 weeks of age, there was a non-significant trend towards increased rearing in the CF group (Number of rearings WT: 16.2 ± 1.6 (n = 15); CF: 19.4 ± 2.3 (n = 17); HDA: 13.2 ± 2.3 (n = 21); DKO: 11.1 ± 1.8 (n = 16)). However, the deletion of Hdac6 in CF mice further reduces rearing behavior (p < 0.05 via ANOVA analysis with Newman–Kuels post-hoc test). These data demonstrate that Hdac6 depletion reduces exploratory behavior in CF mice after 8 weeks of age and may also indicate an increase in anxiety-like behavior in the absence of Hdac6.

#### Open field

A second method to analyze anxiety-like behavior in rodents is the open field test^28^. Mice were placed in the center of this box and their movement, location preference, and behavior was recorded for five minutes. Anxious mice are expected to travel less distance, preferring to be located near a wall and demonstrating less exploratory behavior such as stretching.

#### Distance traveled

**Figure 3:**
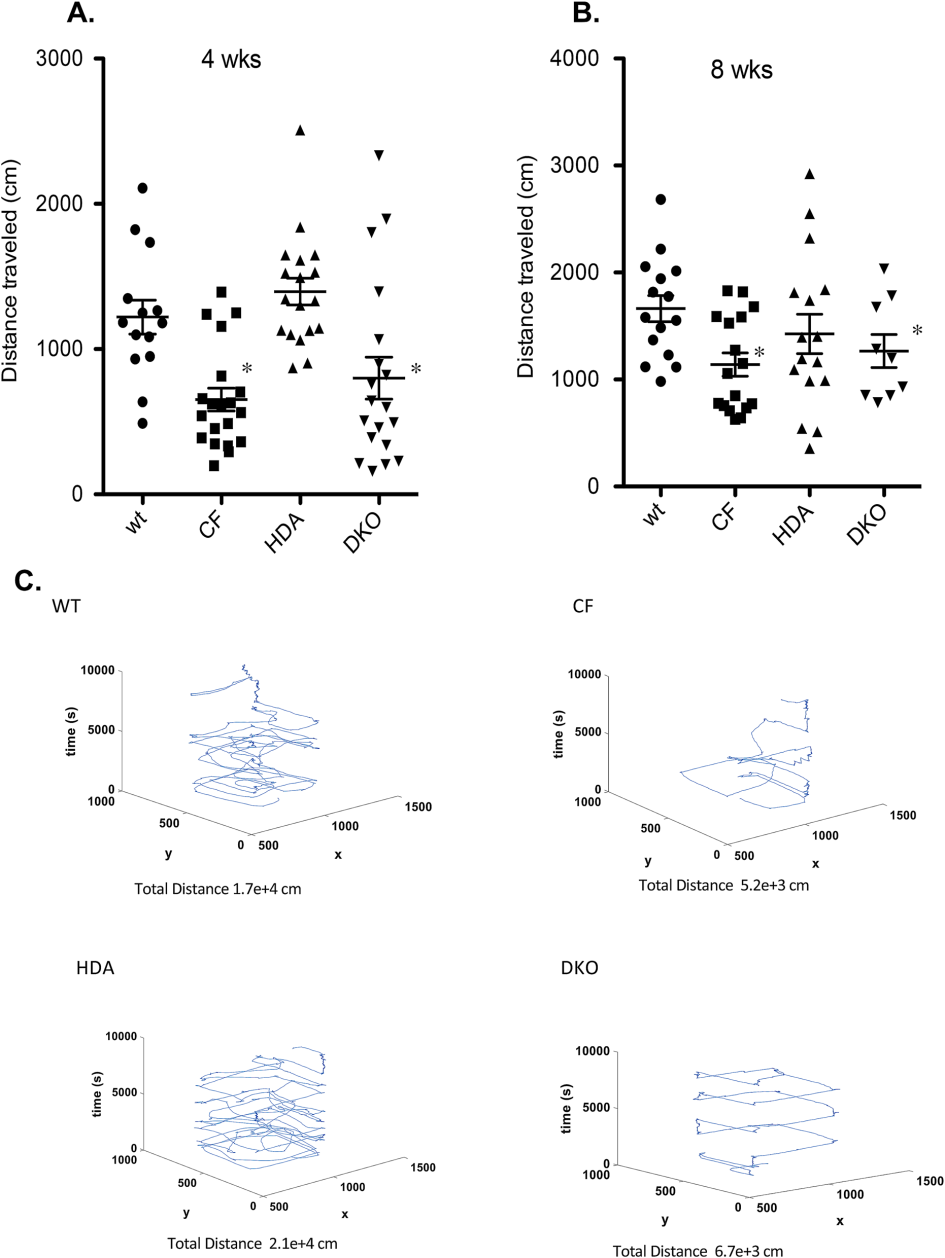
Total distance traveled in open field test. Total distance traveled (cm) in 4-week old (**A**) and 8-week old (**B**) grouped male and female wild type (WT), F508del/F508del (CF), Hdac6-/-(HDA), and CF/HDA (DKO) mice. (**C**) Movement traces generated by IdTracker are shown for WT, CF, HDA, and DKO mice. Significance determine by ANOVA with Newman–Kuels post-hoc test to compare groups; *p < 0.05 compared to WT control group. Replicates for each study are: (**A**) WT = 14, CF = 19, HDA = 18, DKO = 19; (**B**) WT = 15, CF = 17, HDA = 16, DKO = 9.

Both 4- and 8-week old CF mice in the gender combined analysis traveled significantly less distance throughout the open field box than the WT mice (**4wk:** WT: 1220.5 ± 117.3 cm; CF: 652.8 ± 77.9 cm/**8wk:** WT:1662.9 ± 121.9 cm; CF: 1140.1 ± 107.7 cm). Depletion of Hdac6 expression in the DKO mice did not significantly increase distance traveled compared to CF mice (**4wk:** DKO: 800.1 ± 144.7 cm/**8wk:** DKO: 1266.2 ± 154.4 cm) (Fig. 3A,B). Total distance traveled is depicted in Fig. 3C.

#### Location preference

**Figure 4:**
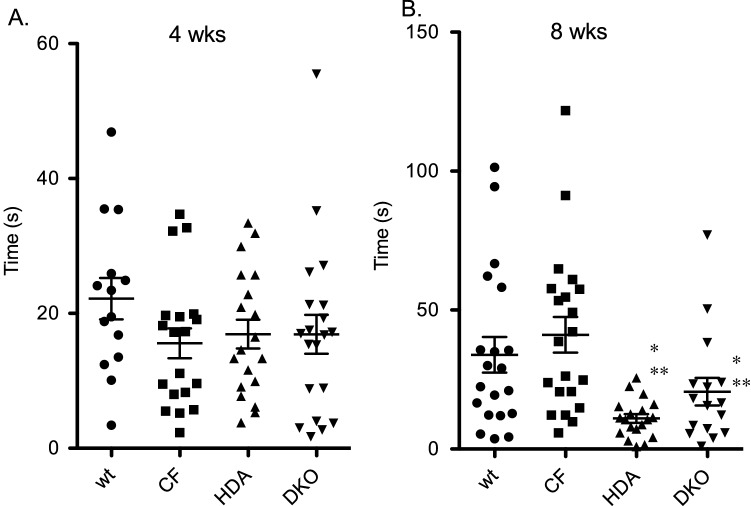
Open area traveling in open field test. Time spent in open-area in 4-week old (**A**) and 8-week old (**D**) grouped male and female wild type (WT), F508del/F508del (CF), Hdac6-/-(HDA), and CF/HDA (DKO) mice. Significance determine by ANOVA with Newman–Kuels post-hoc test to compare groups; *p < 0.05 compared to WT control group, **p < 0.05 compared to CF test group. Replicates for each study are: (**A**) WT = 14, CF = 19, HDA = 19, DKO = 20; (**B**) WT = 20, CF = 21, HDA = 18, DKO = 16.

Mice that are experiencing anxiety would spend more time near a wall and less time in the open middle section of the box. Mice were considered to have entered into the middle of the box when they were at least 10 cm from the wall. There was no difference from in location preference from WT and CF mice at either age (Fig. 4A,B). Depletion of Hdac6 expression in 8-week old mice resulted in decreased open area traveling time suggesting a potential increase in anxiety-like behavior (WT: 33.9 ± 6.4 s; CF: 41.1 ± 6.4 s; HDA: 11.0 ± 1.6 s: DKO: 20.6 + 5.0 s) (Fig. 4B). These data are consistent with the elevated plus maze data in that reduced traveling distance is observed in CF compare to WT mice and that this phenotype is not corrected by Hdac6 depletion.

### Depression-related behavior analysis

#### Tail suspension

**Figure 5:**
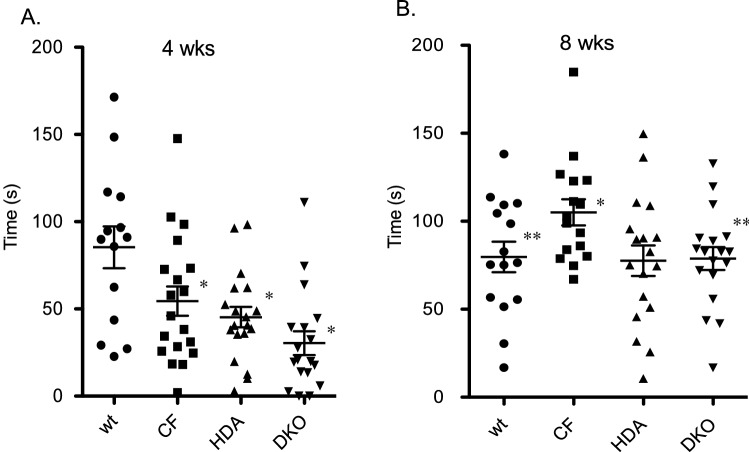
Immobility time in tail suspension test. Time spent immobile (s) during tail suspension test in 4-week old (**A**) and 8-week old (**B**) grouped male and female wild type (WT), F508del/F508del (CF), Hdac6-/-(HDA), and CF/HDA (DKO) mice. Significance determine by ANOVA with Newman–Kuels post-hoc test to compare groups; *p < 0.05 compared to WT control group, **p < 0.05 compared to CF test group. Replicates for each study are: (**A**) WT = 14, CF = 19, HDA = 18, DKO = 18; (**B**) WT = 15, CF = 16, HDA = 18, DKO = 18.

To analyze depression-like behavior in CF mice the tail suspension method was employed^29^. Mice were suspended by their tail for six minutes while being recorded. Mice were considered to show depressive-like behavior when after they are subjected to short-term inescapable stress and present immobility and failure to struggle, swing, climb or shake their body in efforts to free themselves. At 4-weeks of age, CF mice actually exhibit less immobile time compared to WT mice suggesting less depressive behavior (WT: 85.3 ± 12.0 s; CF: 54.5 ± 8.4 s: HDA: 45.2 ± 5.9 s; DKO: 30.3 ± 6.8 s) (Fig. 5A). By 8-weeks of age, however, CF mouse immobility time significantly increases compared to WT controls (WT: 79.7 + 8.6 s; CF: 105.1 + 7.4 s: HDA: 77.6 + 8.6 s; DKO: 78.8 + 6.5 s) (Fig. 5B). Depletion of Hdac6 expression in CF mice restores immobility time to WT levels. Control HDA mice also exhibit WT levels of immobility. These data demonstrate that post-pubertal CF mice develop depression-related behavior that is reversible with the depletion of Hdac6 expression.

#### Hypothalamus acetylated tubulin levels

**Figure 6:**
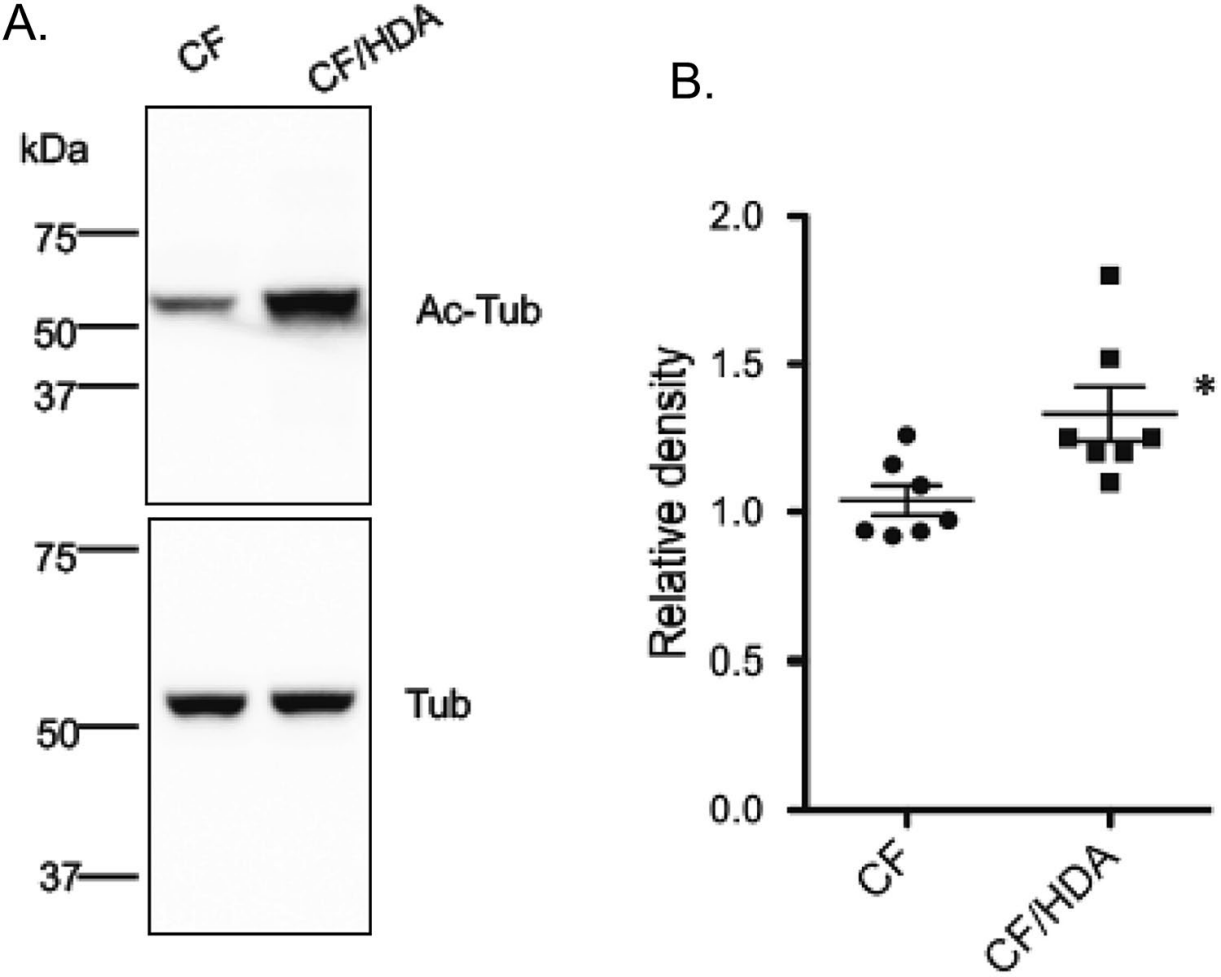
Acetylated tubulin levels in the hypothalamus. Acetylated tubulin (Ac-Tub) levels were assessed in excised hypothalamus from F508del/F508del (CF) and CF/HDA (DKO) mice Western blot and compared to total tubulin (Tub) content. Data are reported as Relative Density (Ac-Tub/Tub). Significance determined by t-test, n = 7 for each group; *p = 0.02.

We have demonstrated previously as a characterization of this mouse model that acetylated tubulin levels were increased in the mouse nasal epithelium of CF/HDA mice compared to CF controls^22^. Since this manuscript is examining neurological effects of Hdac6 depletion, we examined acetylate tubulin content in the hypothalamus of these mice. The hypothalamus was chosen for this analysis since our previous growth studies suggest an impact of HDAC6 on the hypothalamus-pituitary axis in part due to the restoration of IGF-1 production. The hypothalamus has also been shown to be a regulator of depression-like behavior^22,30–32^. There is a significant increase in acetylated tubulin content in the hypothalamus of CF/HDA mice compared to CF mice as determined by Western blot (Fig. 6). These data help characterize the model used in this study and show a direct impact on neuronal tissue regulation by HDAC6.

## Discussion

Anxiety and depression are significant clinical manifestations that adversely affect CF patient quality of life^6–8^. It has been shown that CF mouse models also exhibit anxiety-like behavior qualities suggesting that there is a biological, mechanistic reason for behavior manifestations in addition to the stresses of chronic disease that can contribute to anxiety and depression^13^. We have previously demonstrated that depletion of Hdac6 expression in CF mice improve responses to bacterial challenge as well as growth to more WT levels^22,23^. Growth restoration included both increased weight gain due to increased fat deposition, but also improved linear growth^22^. Linear growth was accompanied by an increase in insulin-like growth factor-1 (IGF-1) production in the DKO mice. The combination of increased linear growth and elevated IGF-1 production point strongly to an effect of Hdac6 depletion in CF mice on the hypothalamus-pituitary axis. Since Hdac6 depletion is having an effect on neurological signaling in CF, coupled with previous reports that HDAC6 inhibition leads to reduced depressive behavior in mice^26^, the hypothesis tested in this manuscript is that Hdac6 depletion would improve anxiety-like and depressive-like behavior in CF mice.

Both the elevated plus maze and open field tests reveal reduced total distance traveled and reduced time in either open arm traveling or open area traveling. These data can be interpreted as increased anxiety. However, this interpretation is complicated since activity levels of CF mice in both assays are reduced as determined by total distance traveled. CF mice display higher resting energy expenditure and can result in less active mice^33,34^. This reduction in total activity is not changed in anyway by the depletion of Hdac6 expression. Neither age nor gender had an impact on these outcome measures. Though a previous study has demonstrated increased anxiety-like behavior in a CF mouse model^13^, the significant change in activity levels makes it difficult to reach a definitive conclusion in this study on anxiety.

In contrast to the anxiety outcome, depressive-related behaviors in CF mice are evident as determined by the tail-suspension assay. When examined at 4-weeks of age, CF mice display no depression-like behavior. However, the phenotype develops over time and is clearly evident by 8-weeks of age. Since reduced activity level is seen at both 4- and 8-weeks of age, it is unlikely that the observed depression-like behavior that develops in CF mice is due to these parameters. Similarly, depletion of Hdac6 in CF mice reverses depression observed at 8-weeks of age but has no influence on activity levels at that age. These data strongly suggest that depression-like behavior develops near puberty in this CF mouse model and that the mechanism involves in part Hdac6-dependent regulation. The apparent change in behavior and responsiveness to Hdac6-depletion that occurs near mouse puberty highly correlates with our previous report demonstrating that Hdac6-depletion restores post-pubertal growth and weight gain in CF mice^22^.

Our study does not address specific mechanisms associated with the correction of depression-like behavior in CF mice, however, earlier studies have addressed the question. Singh et al. have demonstrated that HDAC6 inhibitors disrupt tubulin interactions with Gα_s_ subunit of G-protein coupled receptors (GPCRs), releasing it from lipid rafts and increasing interactions with adenylate cyclase resulting in increased cAMP production, phosphor-cAMP receptor element binding protein (pCREB) levels, and brain derived neurotrophic factor (BDNF) expression^35^. Dissociation of Gα_s_ from lipid rafts also occurs with other known anti-depressants through different mechanisms^35^. Another potential mechanism of action is the anti-inflammatory effect of HDAC6 inhibition^23^. Inflammation has been correlated with depression in a number of studies^35–38^, therefore, alleviating inflammatory signaling may be a mode of action of Hdac6 depletion. We have shown specifically in CF mouse models that depletion of Hdac6 results in reduced inflammation and better clearance of bacteria in airways from infected mice^23^. Several studies have demonstrated the anti-inflammatory benefits of HDAC6 inhibition, with two specifically looking at neuronal inflammation. Song et al. demonstrate that HDAC6 inhibition prevents inflammation including neuroinflammation in LPS treated mice through a p38-dependent pathway^39^. Tseng et al. show the efficacy of Hdac6 depletion in reducing Tau-mediated neuroinflammation^40^. No studies have addressed intrinsic neuroinflammation in the brains of CF animals as of yet, but the anti-inflammatory effects of HDAC6 inhibition cannot be discounted as a potential mechanism of action.

Another aspect of HDAC6 biology that needs to be considered from a mechanistic perspective is the role of HDAC6 in autophagy. Several studies have demonstrated that HDAC6 is a critical component of autophagic aggresome and that its inhibition interferes with the autophagic process^41–43^. Autophagy inhibition is not a likely candidate for the mechanisms of Hdac6 depletion as most studies show that stimulation of autophagy has an anti-depressive effect in a number of conditions^44–46^. However, there are exceptions. The compound silibinin exerts its anti-depressive activity by inhibiting autophagy stimulated by amyloid β1–42 peptide into rats^47^.

In summary, cystic fibrosis is a chronic, difficult to manage disease that induces a broad range of physiological stresses on the patient^6–8^. We hypothesized that while physical stresses from the disorder likely contribute to a higher incidence of anxiety and depression seen in CF patients, there may be more intrinsic biological contributors to the phenotypes. Previous studies in our lab have shown that microtubule stability and vesicle trafficking along microtubules are altered in CF. These results, along with the knowledge that microtubule dysregulation have been implicated in a number of neuronal disorders, led us to examined anxiety and depression behaviors in CF mice. Our data show that loss of CFTR function leads to depression-like behavior in CF mice post-pubertally and that depletion of Hdac6 in CF mice improved the phenotype.

## Methods

### Mice

To create mice without functional CFTR and Hdac6 we crossed mice with the *Cftr* mutation *F508del*^48^ with Hdac6 null mice^49^. Both strains were on a C57Bl/6J background. To decrease the incidence of intestinal obstruction that is common in CF mice, mice were allowed access to sterile water with osmotic laxative, PEG-3350 with electrolytes. All mice were maintained on a 12 h light, 12 h dark cycle at a mean ambient temperature of 22 °C. The Institutional Animal Care and Use Committee (IACUC) of Case Western Reserve University approved all animal protocols. All methods were conducted according to necessary guidelines and established regulations.

### Behavior tests

We used three behavioral assays to test our hypothesis in male and female WT, CF, Hdac6-/- (HDA) and CFTR-/-; Hdac6-/- (DKO) mice; an Elevated Plus Maze (EPM) and an Open Field Box (OF) were used to measure anxiety and a Tail Suspension (TS) assay was used to measure depression-like behavior. Male and female mice from each of the four genotypes (WT, CF, HDA, DKO) were analyzed for anxiety using the elevated plus maze or the open field box and depression using the tail suspension test (specific numbers are provided in figure legends). To account for differences that may be seen due to age we used mice that were 4 and 8 weeks old.

### Elevated plus maze (EPM)

The maze was elevated 51 cm above ground and consisted of four arms each measuring 28 cm long and a center square area measuring 6 cm by 6 cm. Two of the arms were open while two were enclosed on three sides with walls measuring 20 cm high. The maze was oriented in the same direction for every test with the top and bottom arms being open while the left and right arms were closed. Mice were placed in the elevated plus maze in the center section facing the upper, open arm and were allowed to move freely throughout the maze for five minutes while being video recorded from above. Videos were loaded into idTracker, which calculated total distance traveled by each mouse. Location Preference such as open versus closed arm was also analyzed by visually recording entries and time spent in each type of arm using EPMscore^50^. Tests were performed at the same time each day (9–10 AM) in an isolated procedure room with standard room lighting.

### Open field test (OF)

The box consisted of four walls each measuring 41cmX41cm. Mice were placed in the center of an Open Field Box and allowed to move freely throughout the box for five minutes while being recorded. Videos were loaded into idTracker (https://www.idtracker.es/home) which calculated total distance traveled by each mouse. Location preference was also noted by visually recording where the mouse preferred to be (by the wall or in the open) and for how long using EPMscore. Tests were performed at the same time each day (9–10 AM) in an isolated procedure room with standard room lighting.

### Tail suspension (TS)

The Tail Suspension apparatus measured 45 cm wide by 37 cm high by 31 cm deep. Mice were suspended by their tail 7 cm below the top of the box and their behavior was recorded for 6 min. Videos were analyzed using EPMscore^50^ noting the length of escape oriented behaviors such as reaching for the walls, shaking of the body and running. Tests were performed at the same time each day (9–10 AM) in an isolated procedure room with standard room lighting.

### Behavior testing scheme

Mice were allowed to acclimate in the testing room for at least 48–72 h before testing and 24 h were provided between tests. The order of the tests was elevated plus maze, followed by open field, and then tail-suspension. All tests were performed at the same time of day (9–10 a.m.) in the same room so lighting and timing was consistent. Mice were housed with 2–3 mice per cage in accordance with our facility guidelines. Mice are housed in a facility that use a 12-h light, 12-h dark cycle that correspond with night and daylight hours.

### Statistical analysis

Significance for each study was determined by ANOVA with Newman–Kuels post-hoc test to compare groups using Prism Software (PRISM 5.0). Results were considered significant when p ≤ 0.05 in multi group comparisons in reference to WT control group.

## Supplementary information

Supplementary information.

## Data Availability

The datasets generated during and/or analyzed during the current study are available from the corresponding author on reasonable request.
