# Supplementary material for: Alleviation of depression-like behavior in a cystic fibrosis mouse model by Hdac6 depletion
Source: Sci Rep. 2020 Oct 1;10:16278. doi: 10.1038/s41598-020-73298-4 (PMC7530985; doi:10.1038/s41598-020-73298-4)
Supplement: Supplementary file 1 — Supplementary information. [file 41598_2020_73298_MOESM1_ESM.pdf]

Supplementary data

Alleviation of depression-like behavior in a cystic fibrosis mouse model by Hdac6 depletion

Deborah A. Corey, Sharon M. Rymut, and Thomas J. Kelley

Department of Genetics and Genome Sciences

Case Western Reserve University

Cleveland, Ohio

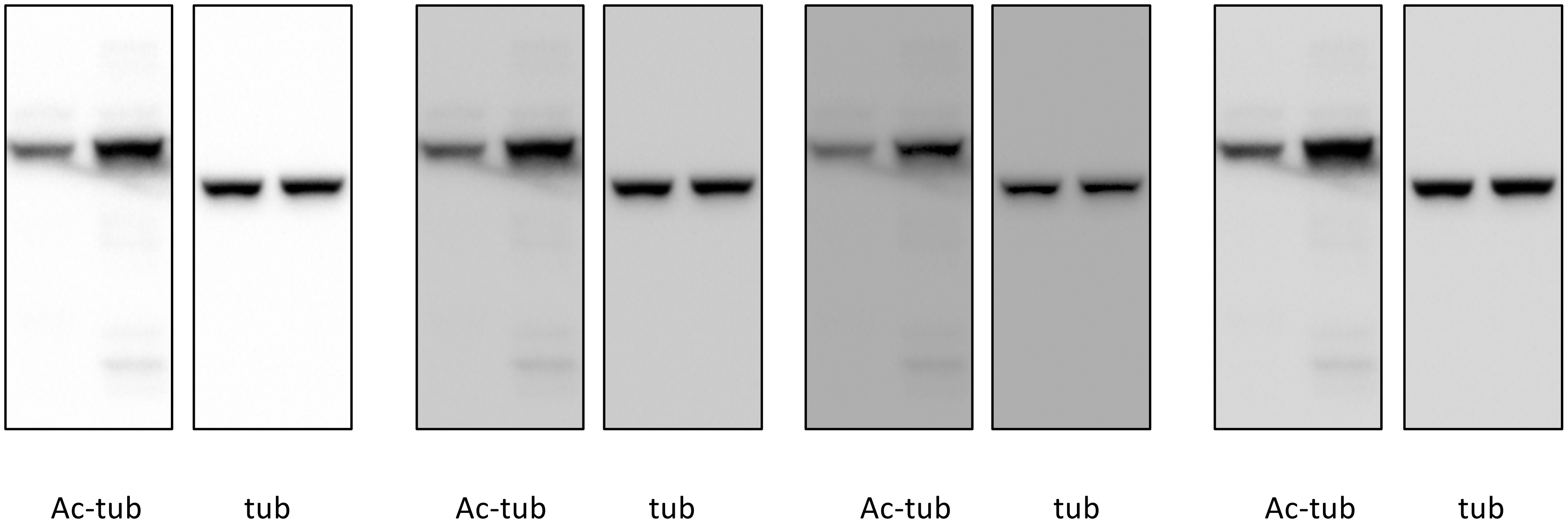

Ac-tub = acetylated tubulin
Tub = tubulin

Supplemental figure 1: Ac-tub and total tubulin Western blots at different exposures
